# Abdominal aortic aneurysm monitoring via arterial waveform analysis: towards a convenient point-of-care device

**DOI:** 10.1038/s41746-022-00717-3

**Published:** 2022-11-04

**Authors:** Mohammad Yavarimanesh, Hao-Min Cheng, Chen-Huan Chen, Shih-Hsien Sung, Aman Mahajan, Rabih A. Chaer, Sanjeev G. Shroff, Jin-Oh Hahn, Ramakrishna Mukkamala

**Affiliations:** 1 Department of Bioengineering, University of Pittsburgh, Pittsburgh, PA USA; 2 Department of Medical Education, Taipei Veterans General Hospital, Taipei, Taiwan; 3 School of Medicine, National Yang Ming Chiao Tung University, Taipei, Taiwan; 4 Division of Cardiology, Taipei Veterans General Hospital, Taipei, Taiwan; 5 Department of Anesthesiology and Perioperative Medicine, University of Pittsburgh, Pittsburgh, PA USA; 6 Department of Surgery, University of Pittsburgh, Pittsburgh, PA USA; 7 Department of Mechanical Engineering, University of Maryland, College Park, MD USA

**Keywords:** Aneurysm, Diagnostic markers, Biomedical engineering

## Abstract

Abdominal aortic aneurysms (AAAs) are lethal but treatable yet substantially under-diagnosed and under-monitored. Hence, new AAA monitoring devices that are convenient in use and cost are needed. Our hypothesis is that analysis of arterial waveforms, which could be obtained with such a device, can provide information about AAA size. We aim to initially test this hypothesis via tonometric waveforms. We study noninvasive carotid and femoral blood pressure (BP) waveforms and reference image-based maximal aortic diameter measurements from 50 AAA patients as well as the two noninvasive BP waveforms from these patients after endovascular repair (EVAR) and from 50 comparable control patients. We develop linear regression models for predicting the maximal aortic diameter from waveform or non-waveform features. We evaluate the models in out-of-training data in terms of predicting the maximal aortic diameter value and changes induced by EVAR. The best model includes the carotid area ratio (diastolic area divided by systolic area) and normalized carotid-femoral pulse transit time ((age·diastolic BP)/(height/PTT)) as input features with positive model coefficients. This model is explainable based on the early, negative wave reflection in AAA and the Moens-Korteweg equation for relating PTT to vessel diameter. The predicted maximal aortic diameters yield receiver operating characteristic area under the curves of 0.83 ± 0.04 in classifying AAA versus control patients and 0.72 ± 0.04 in classifying AAA patients before versus after EVAR. These results are significantly better than a baseline model excluding waveform features as input. Our findings could potentially translate to convenient devices that serve as an adjunct to imaging.

## Introduction

Abdominal aortic aneurysm or AAA (“triple A”) is one of the top 15 leading causes of death in the United States^1^. In this condition, a weakened aortic wall can lead to progressive aortic diameter enlargement and, in some cases, rupture. Aortic rupture is highly morbid and lethal with a mortality rate of up to ~80%^1,2^.

Risk factors for AAA (defined here as a maximal aortic diameter >3.5 cm) include advanced age, male sex, smoking, family history of the condition, and hypertension^1–3^. AAA can be treated via open or endovascular repair. The mortality rate of the surgery can be just 2–3%^1,4^. Surgery is indicated for AAAs >5.5 cm or expanding at a rate >1 cm/year^2,3^. Since most AAAs are asymptomatic, screening and surveillance are essential^1–4^.

Ultrasound permits accurate and safe detection of AAAs with sensitivity/specificity of 94–100%^1–3^. Furthermore, guidelines (e.g., Society for Vascular Surgery) and programs (e.g., Medicare) are in place for one-time screening in specific populations (e.g., men above 65 years^4^ or men and women aged 65–75 years who have ever smoked^2,3^) and for surveillance (e.g., every 12–36 months for AAAs <5 cm^3^). However, ultrasound requires an expert operator, capital equipment, and costs ~$100 per scan^2^. As a result, ultrasound is used in <1–20% of those patients eligible for AAA screening^5^, and AAA diagnosis is often made based on incidental findings when imaging for other reasons or at the time of rupture^1^. About 1.3 years of life are lost for every 10 unscreened patients, which is similar to breast cancer screening^6^. Ultrasound may be underutilized for AAA surveillance as well^5^. The guidelines assume fixed AAA expansion rates, but AAAs can grow in spurts or even shrink over time^7^. The guidelines and programs are also based on the argument that competing causes of death are significant at very old ages (e.g., >80 years)^2^. However, this argument may become less tenable as society ages and the quality of healthcare advances.

Convenient methods for AAA screening and surveillance could thus be impactful^4,5^. However, physical exam via aortic palpation requires skill and is unreliable when the AAA is not large or the patient is not thin, with sensitivity/specificity of 39–68/75%^2^. As a result, key opinion leaders are calling for point-of-care devices to monitor AAA more frequently^4,8^.

**Fig. 1:**
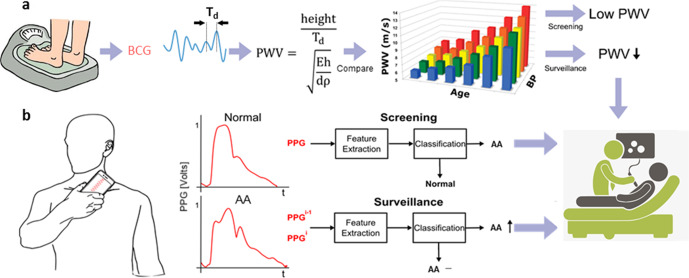
Potential convenient point-of-care devices for abdominal aortic aneurysm (AAA) monitoring via arterial waveform analysis. **a** A sensitive weighing scale for measuring a ballistocardiography (BCG) waveform and detecting aortic pulse wave velocity (PWV)^19^. PWV decreases with increasing AAA diameter (d) per the Moens-Korteweg equation (where E is the elastic modulus of the aortic wall; h, wall thickness; and ρ, blood density) but also increases with blood pressure (BP) and age (bar graph adapted from^24^). **b** A hand-held unit for measuring a photoplethysmography (PPG) waveform from the carotid artery. The waveform shape during systole is altered due to the early, negative wave reflection in AAA^13^. Such devices may be best used to indicate if an accurate, but less convenient, ultrasound scan should be ordered.

Our hypothesis is that analysis of arterial waveforms, which could be obtained with a medical office device that is convenient in use and cost, constitutes a non-imaging solution for providing information about AAA size and thus a useful adjunct to imaging by prompting ultrasound. This hypothesis is illustrated in Fig. 1 and grounded in physiology. In particular, arterial waveforms arise as the superposition of forward and backward traveling pressure waves, and AAA alters wave propagation and reflection and thus the observed waveforms. We aim to initially test the hypothesis using noninvasive carotid and femoral blood pressure (BP) waveforms via tonometry and reference maximal aortic diameter measurements via imaging from patients with AAA and using these two noninvasive BP waveforms from the same patients after endovascular repair (EVAR) and from comparable control patients without AAA. Our results demonstrate that this unconventional approach for AAA monitoring is feasible and has potential to be translated to future clinical practice.

## Results

### Patient data

Table 1 summarizes the patient data for study in terms of sample sizes, demographics, risk factors, and basic hemodynamic values. The data were from our existing database^9,10^ and included records from 50 patients with AAA before and 4 weeks after EVAR and 50 control patients for a balanced set, with 17 of the 50 AAA patient records also having data 3 years post-EVAR. The AAA and control patients were similar but not perfectly matched with some differences including body surface area (BSA), smoking, and heart rate (HR). All patients had an ankle-brachial index (ABI) > 0.9 to generally exclude peripheral arterial disease (PAD). While PAD is more common in diabetes, only one diabetic patient had an ABI > 1.3, which could mask PAD. BP and HR did not change in the AAA patients following EVAR.

**Table 1:** Demographics, risk factors, and basic hemodynamic values of the abdominal aortic aneurysm (AAA) patients before and after endovascular repair (EVAR) and similar control patients.

	Control (*n* = 50)	AAA (*n* = 50 | *n* = 17)	*p*	AAA 4 weeks post-EVAR (*n* = 50 | *n* = 17)	*p*	AAA 3 years post-EVAR (*n* = 17)	*p*
Age (years)	61 ± 15	66 ± 10	0.536				
Male, *n* (%)	45 (90)	45 (90)	1				
Height (cm)	163 ± 7	168 ± 7	>0.001				
Weight (kg)	64 ± 10	68 ± 9	0.0435				
BSA (m^2^)	1.69 ± 0.06	1.73 ± 0.1	>0.001				
Diabetes, *n* (%)	6 (12)	8 (16)	0.569				
Smoking, *n* (%)	10 (20)	27 (54)	>0.001				
ABI (unitless)	1.1 ± 0.1	1.1 ± 0.1	0.47				
Maximal aortic diameter (cm)	—	5.3 ± 1.3	—	—	—	—	—
SBP (mmHg)	137 ± 16	131 ± 17 | 129 ± 17	0.135	126 ± 16 | 129 ± 21	0.48 | 0.84	132 ± 17	0.58
DBP (mmHg)	80 ± 10	78 ± 11 | 77 ± 13	0.270	73 ± 12 | 73 ± 12	0.28 | 0.54	76 ± 10	0.72
MBP (mmHg)	102 ± 13	99 ± 14 | 99 ± 15	0.300	94 ± 14 | 94 ± 15	0.28 | 0.91	99 ± 13	0.92
HR (bpm)	74 ± 11	67 ± 14 | 69 ± 17	0.004	70 ± 13 | 72 ± 16	0.20 | 0.53	67 ± 11	0.70

The two-sided *p*-values were obtained through comparisons between the corresponding values in the two non-*p* columns to the left using paired *t*-tests.

*BSA* body surface area, *ABI* ankle-brachial index, *SBP/MBP/DBP* systolic/mean/diastolic arm cuff blood pressure, *HR* heart rate.

### Model development and evaluation

We analyzed the patient data to investigate prediction of the maximal aortic diameter from carotid and femoral BP waveform features. Our analysis was based on standard, yet powerful, linear regression.

We sought to develop linear regression models that could predict the maximal aortic diameter over its physiologic range from normal to severely diseased. Although we had the image-based reference maximal aortic diameter measurements for the AAA patients, this measurement was not available for the control patients (see Table 1). Therefore, we leveraged strong pre-knowledge of the quantitative characteristics of the normal aortic diameter to model the reference maximal aortic diameters for the control patients as Gaussian white noise with mean and standard deviation of 2.2 and 0.4 cm^11^ (see Data Analysis subsection for further explanation).

**Fig. 2:**
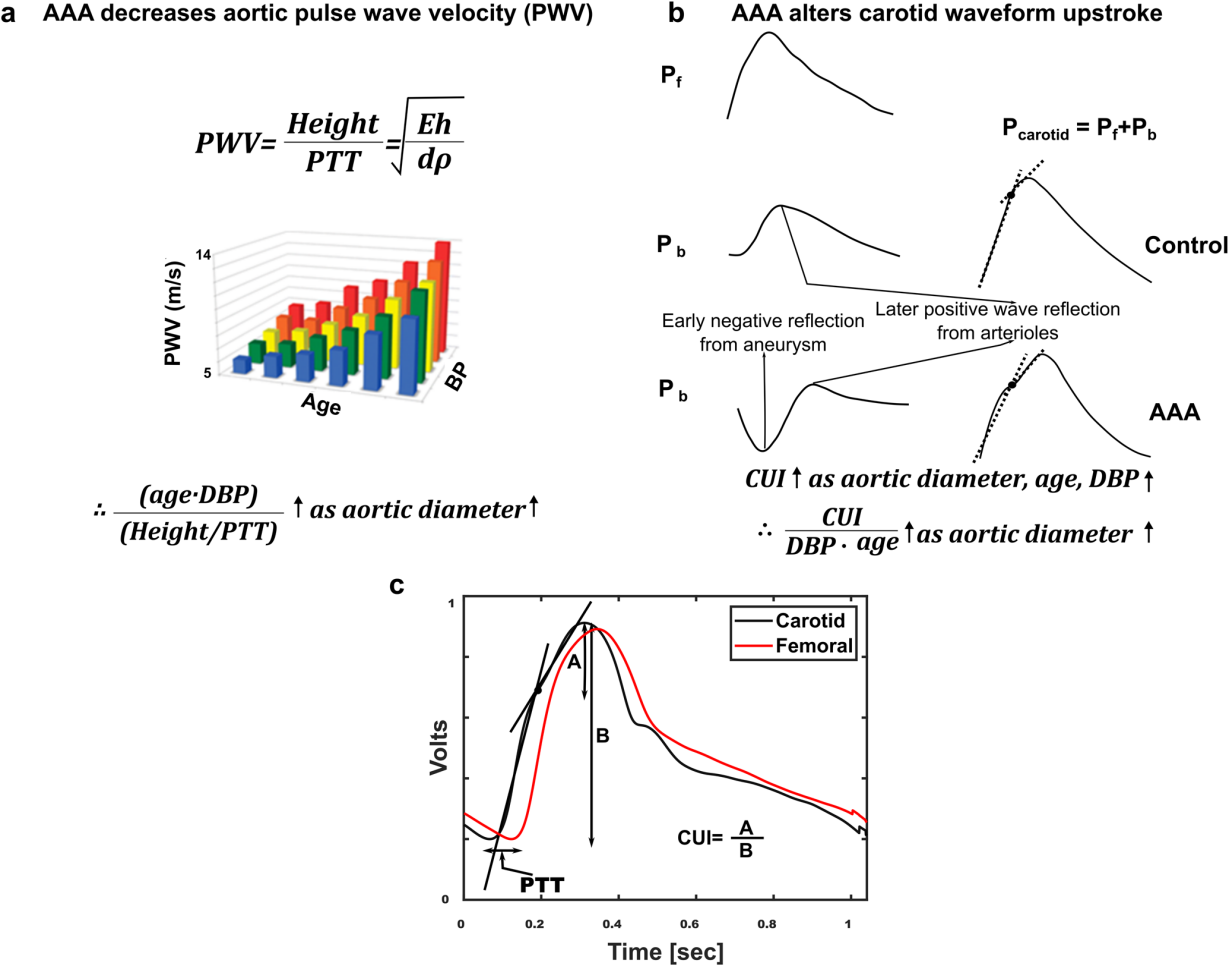
Candidate arterial waveform features based on physiology for indicating the aortic diameter. **a** Pulse transit time (PTT ≈ height/PWV) normalized for age and diastolic BP (DBP) should increase with aortic diameter. **b** A carotid upstroke index (CUI) similarly normalized should increase with AAA diameter due to the early, negative wave reflection. P_carotid_ is the carotid BP waveform; P_f_ and P_b_, forward and backward traveling pressure waves. The waveforms and waves are cartoons. **c** PTT is detected as the foot-to-foot time delay between the carotid and femoral artery waveforms, while CUI is detected from the carotid artery waveform via fitting two lines to its upstroke. The waveforms are ensemble averaged waveform beats of a patient.

**Fig. 3:**
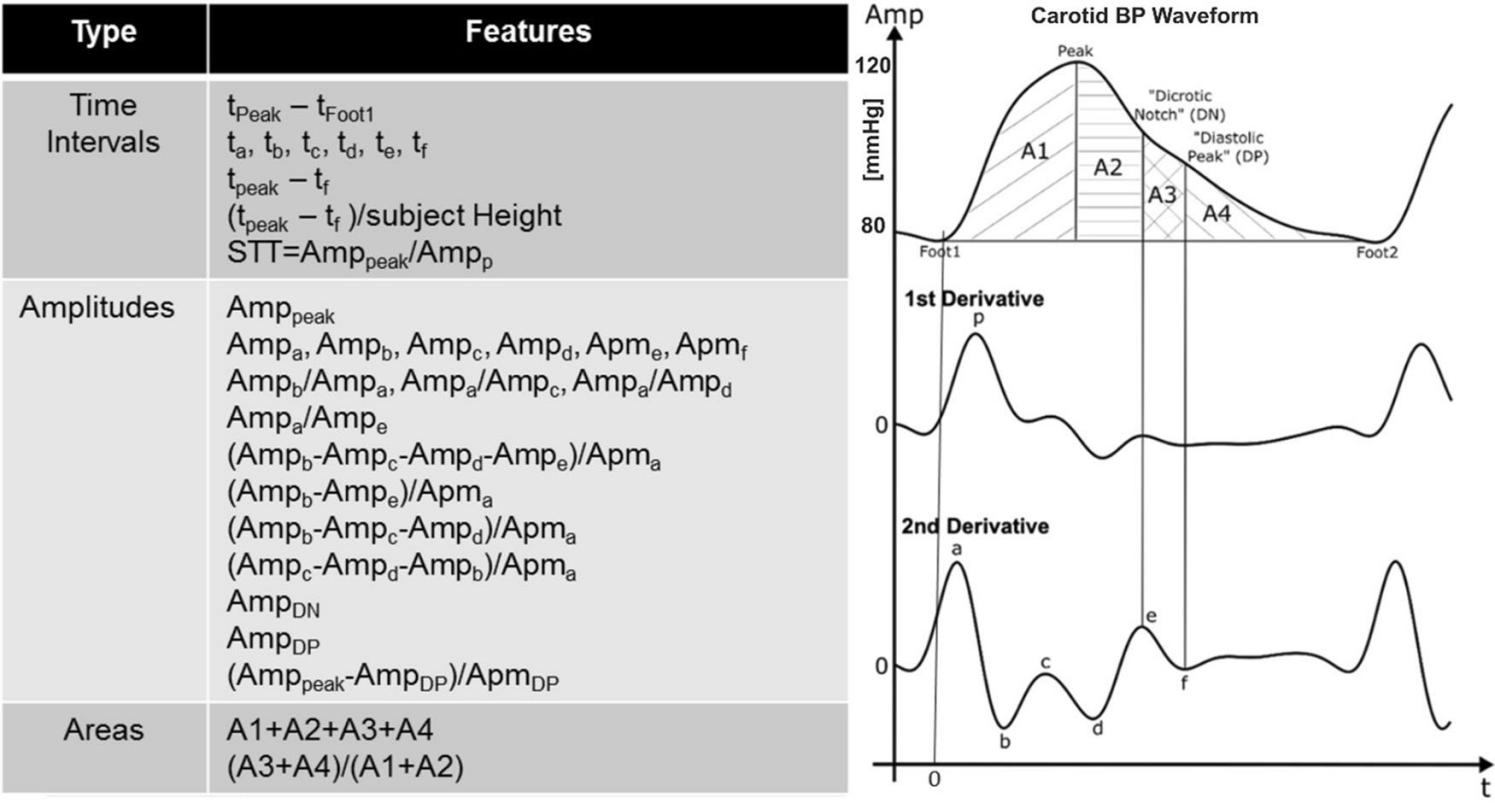
Commonly used features of arterial waveforms to establish a broader candidate set for indicating the aortic diameter^30,31^. These features were extracted from only the carotid BP waveform, since this particular waveform, as perhaps opposed to the femoral BP waveform, could potentially translate to a convenient device (see Fig. 1b).

We extracted features from the waveforms that could potentially translate to a convenient device (see final paragraph of Discussion section) and also considered non-waveform features as model inputs. Figure 2 shows two candidate waveform features that we conceived by invoking physiology, while Fig. 3 shows common waveform features that we used for a broader candidate set. Table 1 lists the candidate non-waveform features (except for the maximal aortic diameter).

We performed feature selection to develop three parsimonious linear regression models for predicting the measured (AAA patients) or modeled (control patients) reference maximal aortic diameter from (i) carotid and femoral BP waveform and non-waveform features (carotid+femoral feature model); (ii) only carotid BP waveform and non-waveform features (carotid feature model); and (iii) non-waveform features alone (baseline model). We employed leave-one-patient-out cross validation to the data from the 50 AAA (pre-EVAR) and 50 control patients to train and test the models. We used the post-EVAR patient data for further and independent testing of the models.

### Developed models

The carotid+femoral feature, carotid feature, and baseline models were stable across the 100 leave-one-patient-out training sets in that 100, 100, and 83% of their regression equations included the same features. Optimal yet representative regression equations for each model trained using all of the data (with the input features adjusted to zero-mean and unit-variance) were as follows:

Carotid+Femoral Feature Model:

1 $$\begin{array}{l}{\mathrm{Maximal}}\,{\mathrm{Aortic}}\,{\mathrm{Diameter}} = 3.79 + 0.78\left( {\frac{{{\mathrm{Area}}\,{\mathrm{Ratio}} \,-\, 0.54}}{{0.21}}} \right)\\ \qquad \qquad\qquad \qquad \qquad \qquad \,\,+\, 0.56\left( {\frac{{\frac{{{\mathrm{Age}} \cdot {\mathrm{DBP}}}}{{{\mathrm{Height/PTT}}}} \,-\, 1701}}{{548}}} \right)\end{array}$$

Carotid Feature Model:

2 $${\mathrm{Maximal}}\,{\mathrm{Aortic}}\,{\mathrm{Diameter}} = 3.79 + 0.96\left( {\frac{{{\mathrm{Area}}\,{\mathrm{Ratio}} - 0.54}}{{0.21}}} \right)$$

Baseline Model:

3 $${\mathrm{Maximal}}\,{\mathrm{Aortic}}\,{\mathrm{Diameter}} = 3.79 - 0.44\left( {\frac{{{\mathrm{HR}} - 70}}{{12}}} \right) + 0.48\left( {\frac{{{\mathrm{BSA}} - 1.71}}{{0.06}}} \right)$$

**Fig. 4:**
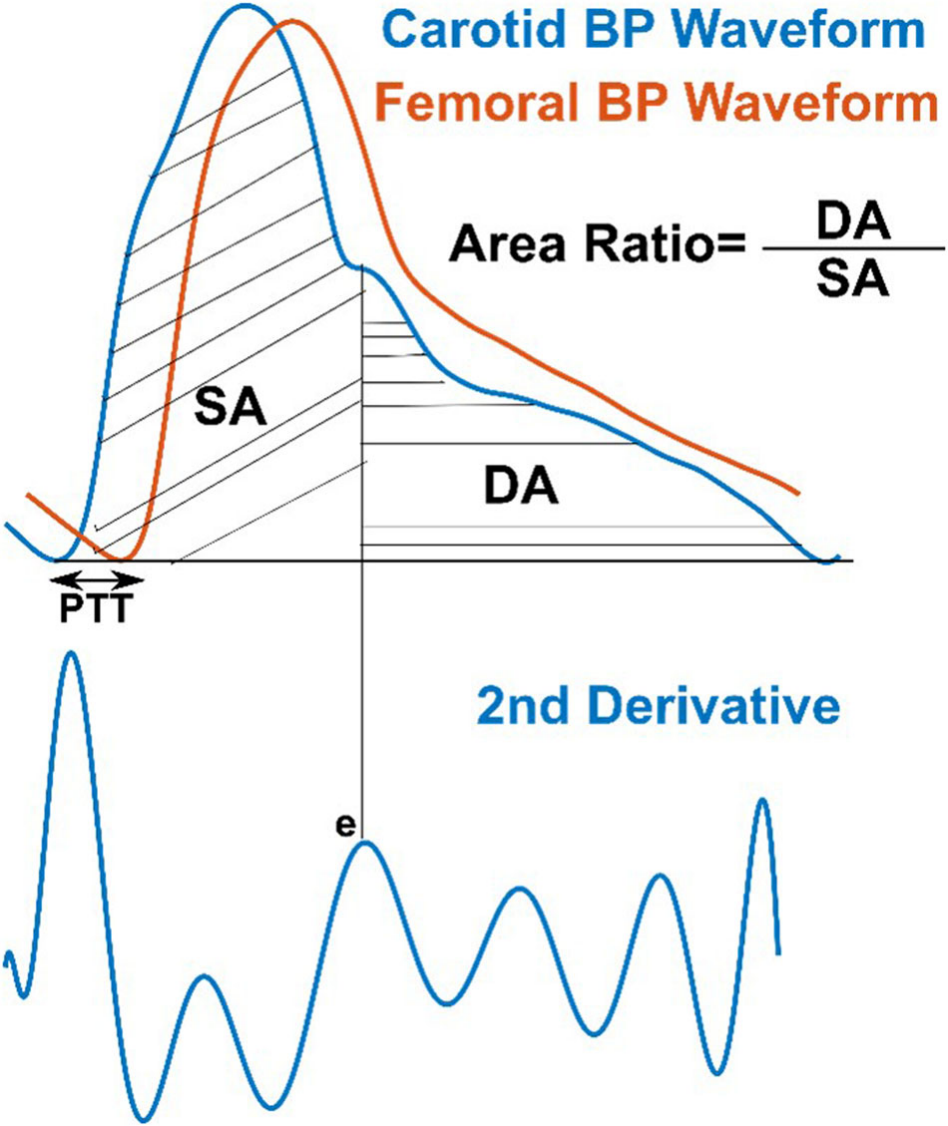
Arterial waveform features selected as inputs to linear regression models for predicting the maximal aortic diameter (see optimal yet representative Eqs. (1) and (2)). The carotid area ratio showed a positive relationship to the maximal aortic diameter, which can be explained in hindsight via the early negative, wave reflection in AAA. The normalized PTT showed a comparably positive relationship to the aortic diameter per physiology (see Fig. 2). All other waveform features (see Figs. 2 and 3) were not helpful.

The carotid+femoral feature model included the area ratio (diastolic area (DA) divided by systolic area (SA) of the carotid artery waveform and unitless) and normalized PTT ((age·DBP)/(height/PTT), where PTT is carotid-femoral pulse transit time in ms, DBP is diastolic BP in mmHg, age is in years, and height is in cm) as the input features. Figure 4 illustrates these waveform features. The two features were comparable in importance based on the relative magnitudes of their model coefficients. The carotid feature model included only the area ratio as the input feature. The input features of the baseline model comprised HR in bpm and BSA in m^2^, which were again significantly different between the AAA and control patients (see Table 1).

### Model prediction of maximal aortic diameter

**Fig. 5:**
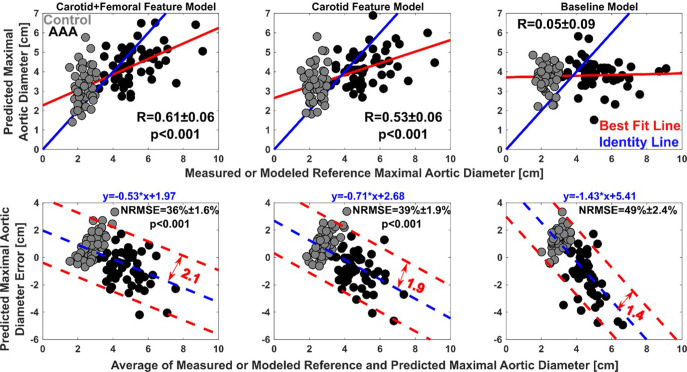
Accuracy results of the 100 leave-one-patient-out maximal aortic diameters predicted by the three models (see optimal yet representative Eqs. (1–3)) for the 100 AAA (before EVAR) and control patients. Correlation plots (top) are predicted versus measured or modeled reference maximal aortic diameters. R (mean ± s.e.m) is correlation coefficient. The two-sided p-values were obtained through comparisons with the baseline model using a common test for dependent data^12^. Bland-Altman plots (bottom) are predicted maximal aortic diameter errors versus the average of the predicted and measured or modeled reference maximal aortic diameters. NRMSE (mean ± s.e.m) is the overall normalized-root-mean-squared-error in percent. The two-sided *p*-values were obtained through comparisons with the baseline model using nonparametric bootstrapping for paired data. The black datapoints denote AAA patients with measured reference maximal aortic diameters, while the gray datapoints indicate control patients with modeled reference maximal aortic diameters (see text for explanation).

Figure 5 illustrates correlation and Bland-Altman plots of the 100 leave-one-patient-out predicted maximal aortic diameters of the three models versus the measured maximal aortic diameters for AAA patients (black) and the modeled reference maximal aortic diameters for the control patients (gray). Note that the control patient datapoints are included in these plots, even though measured reference maximal aortic diameters were unavailable, in order to fairly show the limitations of the models (as discussed below in this paragraph) and their capabilities. The correlation coefficient (R; mean ± s.e.m) between the maximal aortic diameters predicted by the baseline model and the measured or modeled maximal aortic diameters was just 0.05 ± 0.09, whereas the corresponding correlation coefficients for the carotid+femoral feature and carotid feature models were 0.61 ± 0.06 (*p* < 0.001; common test for dependent data^12^) and 0.53 ± 0.06 (*p* < 0.001; common test for dependent data^12^). The overall normalized-root-mean-squared-error (NRMSE; mean ± s.e.m) of the predicted maximal aortic diameters of the baseline model was 49 ± 2.4%, while the corresponding errors for the carotid+femoral feature and carotid feature models were 36 ± 1.6 (*p* < 0.001; nonparametric bootstrapping for paired data) and 39 ± 1.9% (*p* < 0.001; nonparametric bootstrapping for paired data). However, the two waveform feature models as well as the baseline model did sometimes clearly overestimate the maximal aortic diameters for the control patients and did generally underestimate the maximal aortic diameter for the AAA patients. This overestimation and underestimation can be seen in the correlation plots directly and in the Bland-Altman plots via significant inverse correlation between the predicted maximal aortic diameter errors and the average of the predicted and measured or modeled reference maximal aortic diameters.

**Fig. 6:**
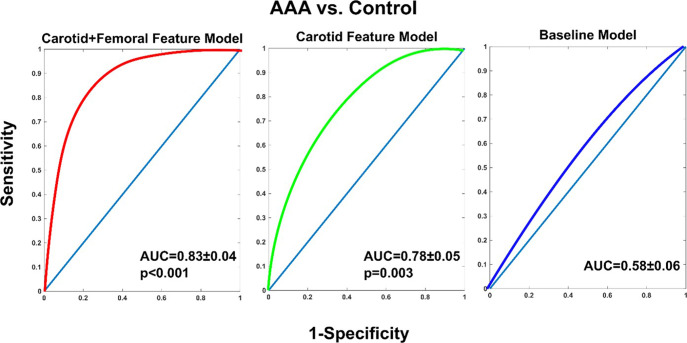
Classification results of the 100 leave-one-patient-out maximal aortic diameters predicted by the three models for the 100 AAA (before EVAR) and control patients. Receiver operating characteristic (ROC) curves for classifying AAA versus control patients using the predicted maximal aortic diameters. AUC (mean ± s.e.m) is ROC area under the curve. The two-sided p-values were obtained through comparisons with the baseline model using the Hanley–McNeil test for paired data.

Figure 6 shows receiver operating characteristic (ROC) curves for classifying AAA versus control patients using the same 100 leave-one-patient-out predicted maximal aortic diameters of the three models. The ROC area under the curve (AUC; mean ± s.e.m) for discriminating the two patient groups via the baseline model was 0.58 ± 0.06 and thus similar to a coin flip, whereas the corresponding AUCs for the carotid+femoral feature and carotid feature models were 0.83 ± 0.04 (*p* < 0.001; Hanley–McNeil test for paired data) and 0.78 ± 0.05 (*p* = 0.003; Hanley–McNeil test for paired data). Furthermore, at 75% specificity, the ROC curves indicated that the carotid+femoral feature and carotid feature models yielded 83% and 66% sensitivities, which compare favorably to the 39–68% sensitivity of aortic palpation at the same specificity^2^.

These results indicate that the carotid+femoral feature and carotid feature models, but not the baseline model, offered value in predicting the maximal aortic diameter.

### Model prediction of EVAR-induced changes in maximal aortic diameter

**Fig. 7:**
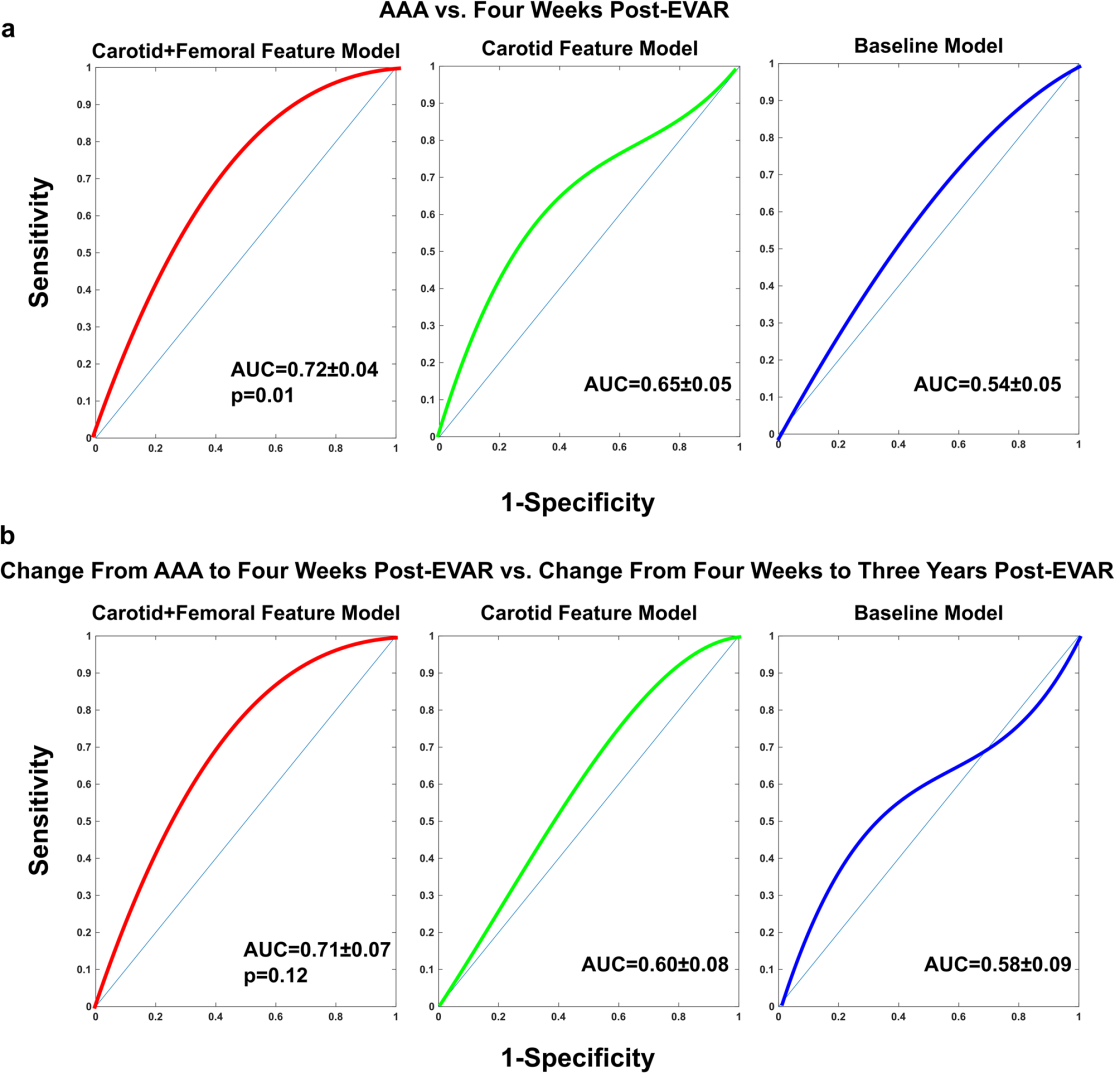
Classification results of out-of-training maximal aortic diameters predicted by the three models for the AAA patients before and after EVAR. **a** ROC curves for classifying the AAA patients before versus 4 weeks after EVAR using the 100 predicted maximal aortic diameters. **b** ROC curves for classifying the change from before to 4 weeks after EVAR versus the change from 4 weeks to 3 years post-EVAR using the 34 available predicted maximal aortic diameter changes. The two-sided *p*-values were obtained through comparisons with the baseline model using the Hanley–McNeil test for paired data.

Figure 7a illustrates ROC curves for classifying AAA patients before versus 4 weeks after EVAR using 100 out-of-training predicted maximal aortic diameters of the three models. Figure 7b shows ROC curves for classifying the change from before to 4 weeks after EVAR versus the change from 4 weeks to 3 years post-EVAR using 34 out-of-training predicted maximal aortic diameter changes of the three models. The two ROC AUCs for discriminating the surgery via the baseline model were 0.54 ± 0.05 and 0.58 ± 0.09, whereas the corresponding AUCs for the carotid+femoral feature model were 0.72 ± 0.04 (*p* = 0.01; Hanley–McNeil test for paired data) and 0.71 ± 0.07 (*p* = 0.12; Hanley–McNeil test for paired data). The AUCs for the carotid feature model were 0.65 ± 0.05 and 0.60 ± 0.08; these values were not significantly different from those of the baseline model (Hanley–McNeil tests for paired data). The results indicate that the carotid+femoral feature model, but not the baseline model, afforded value in predicting EVAR-induced changes in the maximal aortic diameter.

### Model input waveform features and BP waveforms of individual patients

**Fig. 8:**
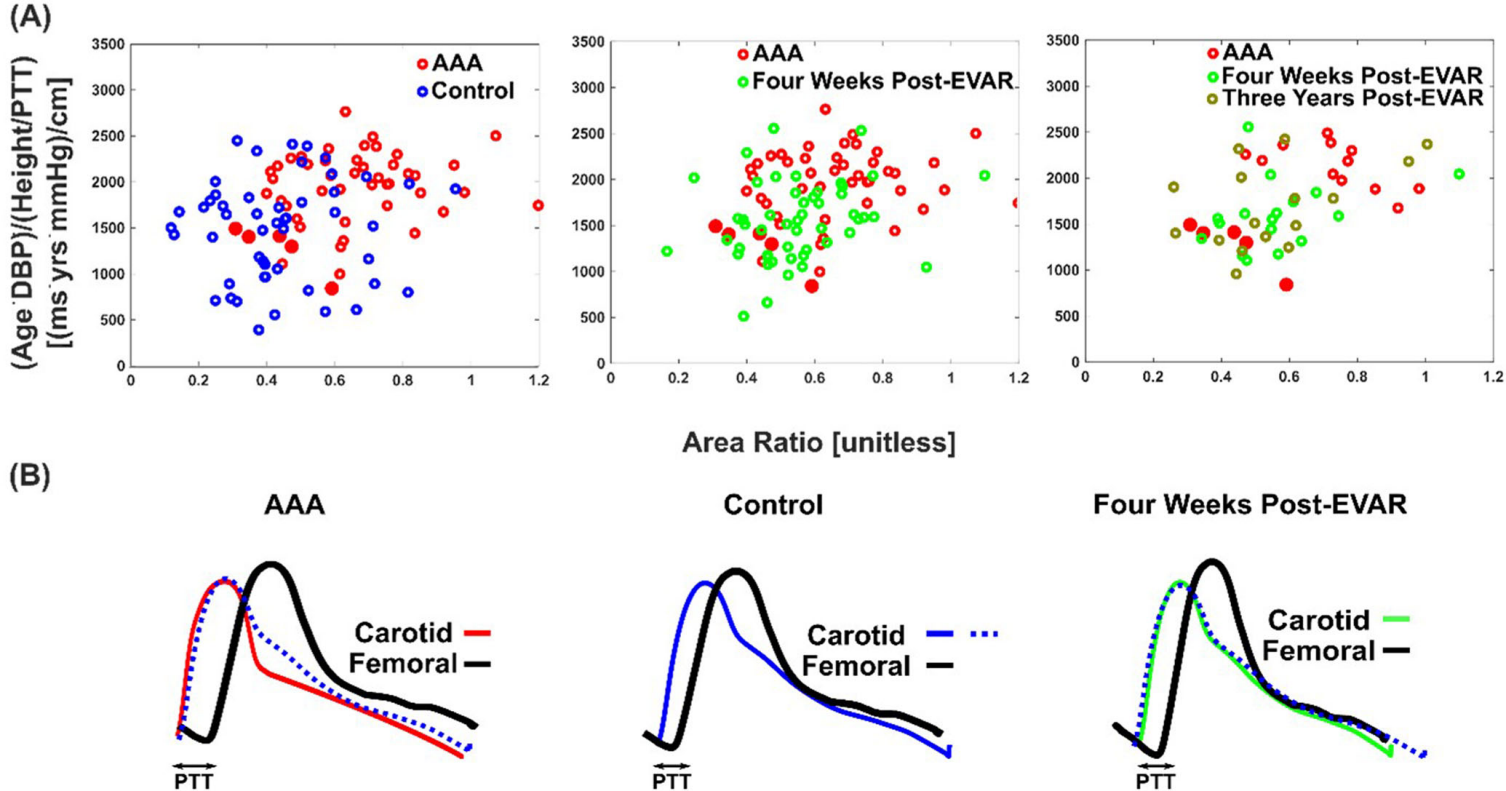
Individual patient results and examples. **A** Plots of normalized PTT versus the area ratio per patient for each of the three classification tasks (see Figs. 6 and 7). The red filled circles are five of the seven AAA datapoints that overlap most with the control and post-EVAR datapoints and thus least discriminating. **B** Visually apparent examples of the differences in the carotid and femoral artery waveforms from AAA (before and after EVAR) and control patients. The waveforms are ensemble averaged waveform beats of a patient. The solid blue waveform beat in the center panel was redrawn as a dashed blue waveform beat in the left and right panels to facilitate comparisons.

Figure 8A illustrates plots of the normalized PTT against the area ratio per patient for the AAA versus control patients (left panel of the figure), AAA patients before versus 4 weeks after EVAR (center panel of figure), and AAA patients before versus 4 weeks after EVAR versus 3 years after EVAR (right panel of figure). Consistent with the quantitative ROC curve results of Figs. 6 and 7a, the separation between the AAA and control patient datapoints (left panel of Fig. 8A) qualitatively appeared greater than the separation between the AAA and 4 weeks post-EVAR datapoints (center panel in Fig. 8A). While there was noticeable separation between the fewer AAA and 4 weeks or 3 years post-EVAR datapoints, this subset of the data happened to include five of the least discriminating AAA datapoints overall (see five filled red circles, which along with two open red circles overlapped most with the control and 4 weeks post-EVAR datapoints in the left panels of Fig. 8A and were thus least discriminating in this way).

Figure 8B illustrates more extreme examples of the carotid and femoral artery waveforms from AAA (before and after EVAR) and control patients. The carotid waveform appeared narrower/sharper with AAA as opposed to wider/rounder without AAA (for a higher area ratio), while the time delay between the carotid and femoral waveforms was longer with AAA (for a higher normalized PTT).

## Discussion

AAAs are lethal but treatable, yet they are substantially under-diagnosed and under-monitored. So, there is profound need for new AAA screening and surveillance devices that are convenient in use and cost. Our hypothesis is that analysis of arterial waveforms, which could be obtained with such a device, can provide information about AAA size (see Fig. 1). We aimed to initially test this physiology-grounded hypothesis using noninvasive carotid and femoral BP waveforms and reference image-based measurements of the maximal aortic diameter from AAA patients and using these two noninvasive BP waveforms from the same patients 4 weeks/3 years following endovascular repair (EVAR) and from similar control patients. After selecting patient data (see Table 1) from our existing database, we developed linear regression models for predicting the maximal abdominal aortic diameter from waveform features (see Figs. 2 and 3) or other information (see Table 1). We then evaluated these models in terms of their ability to predict the maximal aortic diameter value and changes induced by the EVAR.

This study was inspired by our earlier AAA investigation^10^. The aim of that study was to show that carotid-femoral pulse wave velocity (PWV ≈ height/PTT) and carotid augmentation index are not suitable markers of large artery stiffness and wave reflection in AAA patients. Also in contrast to the present study, we did not previously perform multivariable analysis, assess individual patient differences, and analyze the 3 years post-EVAR data. While we did use the same patient database in the earlier study, the overlap of patient data between the two studies was less than half.

We developed three parsimonious and stable models for predicting the maximal aortic diameter from waveform or non-waveform features (see Eqs. (1–3) and Fig. 4). The carotid+femoral feature model included the carotid area ratio and normalized PTT as comparably important input features, while the carotid feature model included only the area ratio as the input feature. The normalized carotid upstroke index (CUI) that we formulated on the basis of an early, negative wave reflection in AAA^13^ (see Fig. 2) did not appear in either model. In hindsight, the area ratio may better quantify this phenomenon. With AAA growth, the negative reflected wave during early systole (waveform upstroke) or even late systole (initial downstroke) should progressively reduce SA without impacting DA as much (see Fig. 2) and thereby increase the area ratio (DA/SA). Note that, while age and BP could likewise be confounders, post-hoc normalizations of the area ratio were not fruitful. The positive model coefficients for the area ratio are consistent with this explanation. In post-hoc analysis, likewise unnormalized CUI did reveal ability to quantify the early, negative wave reflection in AAA, especially in terms of being higher before versus after EVAR (results not shown). However, the area ratio was better in the training data and thus preferred. Similarly, the positive model coefficient for the normalized PTT is congruent with the Moens-Korteweg equation for relating PWV to vessel diameter (see Fig. 2). (See Methods section for more detailed physiologic explanations of the features.) The input features of the baseline model were HR and BSA, which were different between the imperfectly matched AAA and control patients (see Table 1). However, HR, BSA, or any other non-waveform feature did not appear in the carotid+femoral or carotid feature model, thereby suggesting, prior to cross validation, the greater importance of arterial waveform features.

We indeed found from cross validation that only the carotid+femoral feature and carotid feature models were useful in predicting the maximal aortic diameter values of the AAA (pre-EVAR) and control patients (see Figs. 5 and 6). The maximal aortic diameter predictions of carotid+femoral feature and carotid feature models showed appreciable correlations with the measured or modeled reference maximal aortic diameters and yielded ROC AUCs of 0.83 ± 0.04 and 0.78 ± 0.05 in classifying the two groups. Notably, these ROC-based “screening” results indicated greater sensitivity than aortic palpation at a specificity of 75% (83 and 66% for carotid+femoral feature and carotid feature models vs. 39–68% for aortic palpation)^2^.

We also found from cross validation that only the carotid+femoral feature model was useful in predicting the maximal aortic diameter changes induced by EVAR (see Fig. 7). The maximal aortic diameter predictions of the model yielded a ROC AUC of 0.72 ± 0.04 in classifying pre- versus 4 weeks post-EVAR. These predicted maximal aortic diameters did correctly decrease in 84% of the patients following EVAR, but the magnitude of the reductions was not consistently large (e.g., >30%; results not shown). The changes in the predicted maximal aortic diameters of the model also yielded a ROC AUC of 0.71 ± 0.07 in classifying the change from before to 4 weeks after EVAR versus the change from 4 weeks to 3 years post-EVAR in the subset of patients with complete study records. The stent graft itself is stiffer than the normal aortic wall and may cause an early, positive wave reflection^10^. Both the stiffness and wave reflection changes should have helped the normalized PTT and area ratio in discriminating the EVAR. Yet, these “surveillance” results were not quite as good as the screening results (also compare left and center panels of Fig. 8A). The reasons could be variability related to the stent grafts (e.g., diverse materials) or that the models were completely blinded to the post-EVAR data as opposed to blinded in a leave-one-patient-out sense. Also note that the patient subset with 3 years post-EVAR data was small (n=17) and an unfortunate representation of all patients (see five of the least discriminating AAA datapoints (filled red circles) in left panel also in right panel of Fig. 8A).

In general, our findings indicate that, in AAA, the carotid artery waveform appears narrower/sharper and the time delay between carotid and femoral artery waveforms appears longer (see Fig. 8B). While these differences may not always be obvious visually, they could be ascertained computationally as proposed herein. As also suggested by the individual patient data (see Fig. 8A, B), the waveform feature models had modest capacity in classifying the post-EVAR versus control patients (ROC AUCs of 0.64–0.65), which may further verify the models.

The carotid+femoral feature model performed better than the carotid feature model, especially in tracking the EVAR-induced maximal aortic diameter changes, due to the inclusion of the normalized PTT as a second input feature. An earlier study showed that carotid-femoral PWV was inversely correlated with the maximal aortic diameter in earlier stage AAA patients than those studied herein^14^. Hence, normalized PTT may be a consistently important feature throughout AAA growth. Another previous study showed that advanced machine learning could compute carotid-femoral PWV from the carotid artery waveform alone based on about 5000 Framingham Heart Study participants^15^. However, the PWV computation formula was not reported, and that study did not include AAA patients. Nevertheless, the two past studies together with our findings suggest the possibility that a carotid feature model could offer greater value in AAA monitoring than what was reported herein.

The earlier work motivated us to explore machine learning beyond linear regression. We tried various methods including logistic regression for classification without maximal aortic diameter prediction, nonlinear regression via the addition of quadratic terms comprising the normalized PTT and area ratio, neural networks with single hidden layers for establishing more general nonlinear relationships between the maximal aortic diameter and waveform and non-waveform features, and principal components analysis for feature dimensionality reduction. However, we were not able to improve upon our linear regression results. The likely reason is that our cohort of 100 patients with one to three study visits per patient was not large enough.

One limitation of this study was indeed the patient sample size. With more data, it may have been possible to improve the study results through more powerful analytical tools for identifying more accurate, nonlinear equations as an example. It would have also been possible to employ a distinct dataset for testing, which is preferred over leave-one-patient-out cross validation. However, the aim of our study was simply to show initial proof-of-concept that arterial waveform analysis can provide information about AAA size. Another study limitation was that the patient data did not include reference image-based maximal aortic diameter measurements for the control patients. To effectively leverage powerful linear regression, we modeled the reference maximal aortic diameter measurements for the control patients using Gaussian white noise of known statistics. Due to the error introduced in the reference values, the correlation coefficients and Bland-Altman errors (see Fig. 5) could actually be higher and lower, respectively, than reported. However, any differences may only be minor, because the error of the linear regression models in predicting the maximal aortic diameter was likely larger than the error in the reference values due to the relatively narrow range of normal maximal aortic diameters^11^. Also note that the classification tasks and results (Figs. 6 and 7) did not require quantitative reference measurements. A third limitation was the exclusion of patients with PAD and other aortic aneurysms. We omitted PAD, which can accompany AAA, as it too can alter carotid and femoral artery waveforms (via a late, positive wave reflection). However, with additional patient data, it may be feasible to accommodate PAD (e.g., by including the ABI as a model input feature). We eliminated thoracic aortic aneurysms, because their diameters are not comparable to those of AAAs. However, in principle, arterial waveform analysis is applicable to any aortic aneurysm. It is also important to note that physical exam is not an option for detecting aortic aneurysms above the abdomen.

Our study suggests potential point-of-care devices that are convenient in use and cost for providing information about AAA size. One such device is a weighing scale (see Fig. 1a). A sensitive weighing scale or force plate can measure the ballistocardiography (BCG) waveform^16–18^. We previously showed that the BCG waveform in the head-to-toe direction arises as a linear combination of BP waveforms from the ascending aorta, aortic arch, and descending aorta and that the time interval between the I-wave onset and J-wave peak of the BCG waveform is indicative of aortic PTT^19^. Another possible device is a hand-held unit with a photoplethysmography (PPG) sensor for measuring blood volume oscillations (see Fig. 1b). Since the larger carotid artery may be mainly elastic, PPG and tonometry waveforms at this site may appear similar in morphology^20^. Although these devices may show low positive predictive value in the general population, they could be effectively used by caregivers in the primary care setting for screening only patients at high risk for AAA (i.e., those eligible for screening and others such as people with a family history of AAA) and surveillance of patients with known AAA before and perhaps even after surgery to detect endoleaks. If the device results turn out positive, then an ultrasound scan would follow. In this way, a convenient point-of-care device for AAA monitoring via arterial waveform analysis could potentially help reduce AAA mortality. Pursuit of such devices and validation using external or prospective testing data may be worthwhile.

## Methods

### Overall approach

We selected AAA and control patient data from a database that we had previously constructed for other purposes. We analyzed the data to (i) develop models for predicting the maximal abdominal aortic diameter using tonometric carotid and femoral BP waveforms and (ii) evaluate these models in terms of their ability to predict the maximal aortic diameter value and changes induced by EVAR.

### Patient data

We collected data from patients with and without aortic aneurysms at Taipei Veterans General Hospital from 2010 to 2017. These patient studies conformed to the principles outlined in the Declaration of Helsinki and were approved by the Hospital’s IRB, and each participant provided written, informed consent. A subset of the patient database and complete study procedures are presented elsewhere^9,10^.

Briefly, the database consists of records from >200 patients with mainly AAAs but also thoracic aortic aneurysms or other aortic aneurysms (i.e., aortic dissection or multiple aortic aneurysms). Many of the patient records comprise data before and 4 weeks after EVAR with a stent graft, and some records even had follow-up data 3 years post-EVAR. The database also includes records from >200 patients without aneurysmal disease (“controls”). The data per patient and study visit include: (1) carotid and femoral artery tonometry waveforms as well as ECG waveforms at a sampling rate of >250 Hz (VP-2000 system, Colin, Japan); (2) BP values via an oscillometric arm cuff; and (3) patient information including demographics and risk factors. The data from the AAA patients before EVAR also included the reference maximal vessel diameter via ultrasound or CT. The University of Pittsburgh’s IRB declared that this secondary analysis of the existing, de-identified patient database met the regulatory requirements for exempt research (STUDY22020004).

For this study, our data inclusion criteria were (i) patients with AAA and data before and 4 weeks following successful EVAR with the stent graft (which restores the uniform cross-sectional area along the abdominal aorta^10^) and (ii) control patients without any aortic aneurysm. Our data exclusion criteria were (i) multiple aortic aneurysms; (ii) PAD as ascertained via an ABI < 0.9, (iii) non-sinus rhythm; and (iv) artifact-contaminated tonometric waveforms based on visual inspection. We also sought to match the AAA and control patients in terms of non-waveform characteristics, especially sex, age, and arm cuff BP. This matching was crucial for mitigating the possibility of obtaining trivial results.

### Data analysis

Our analysis of the patient data comprised the following steps. After preprocessing the arterial waveforms, we extracted a set of candidate waveform features. We then applied stepwise linear regression to develop easy-to-understand models for predicting the maximal aortic diameter from select waveform features plus basic patient information as well as from the non-waveform information alone. We finally employed cross validation to evaluate the predicted maximal aortic diameters in terms of their accuracy against reference values and their ability to classify AAA versus control patients and AAA patients before versus after EVAR.

#### Waveform Preprocessing

We pre-processed the waveforms similar to our prior work^21^. Briefly, we applied a bandpass filter with 0.5–10 Hz passband to each of the tonometric waveforms. We employed ECG-gating to detect the waveform peaks and then the waveform feet using the intersecting tangent method^22^. We determined the time intervals from the leading foot to lagging foot and leading foot to peak and the amplitudes of the peak and lagging foot relative to the leading foot amplitude for each waveform. We selected the five waveform beats with features closest to the median values in the least squares sense and ensemble averaged these waveform beats starting from the leading foot. We aligned the pairs of carotid and femoral waveform beats in time via ECG-gating. We calibrated the representative waveform beats to cuff diastolic and mean BP. This calibration procedure is standard for tonometric waveforms and based on the fact that diastolic and mean BP (but not systolic BP) are similar throughout the larger arteries^23^. In this way, we arrived at noninvasive carotid and femoral BP waveforms for further analysis.

#### Waveform feature extraction

We extracted a set of candidate waveform features that could potentially translate to a convenient device (see Fig. 1). We conceived two of the features by invoking physiology (see Fig. 2) and used common features for a broader set (see Fig. 3).

We formulated the first physiology-based feature as follows (see Fig. 2a, c). The hallmark change of AAA is an increase in aortic diameter (d). AAA may also increase the elastic modulus (E) of the aortic wall^24^ but may have little effect on the average wall thickness (h)^25^. So, according to the Moens-Korteweg equation (PWV = √(Eh/dρ), where ρ is blood density), if d increases more than E, aortic PWV should decrease with AAA growth. We extracted aortic PWV at the level of diastolic BP (DBP) via the standard foot-to-foot time delay between the carotid and femoral artery waveforms (i.e., PTT). However, E and thus PWV also increase with BP and age^26,27^. Hence, we normalized the PWV, or equivalently, PTT as (age·DBP)/(height/PTT). This feature is expected to increase as the aortic diameter increases.

We formulated the second physiology-based feature as follows (see Fig. 2b, c). Normally, the main arterial wave reflection sites are at the level of the arterioles due to the abrupt change in vessel diameter^28^. Because of vessel tapering, the reflection coefficient and thus the reflected wave are positive^28^. However, in AAA, the vessel diameter is larger at some distance from the heart. The increased diameter causes a negative reflection coefficient and reflected wave at a more proximal site^13^. Hence, carotid artery waveforms should differ in shape during systole with AAA growth due to the superposition of an early, negative wave reflection. While the augmentation index of the carotid artery waveform may be used to quantify this difference, it can be difficult to detect due to the use of higher-order derivatives. So, we instead extracted a more robust index as follows. First, we fitted two lines (each with an adjustable slope and intercept) to the carotid BP waveform samples between the leading foot and peak. Then, we identified the waveform amplitude at the intersection of the two fitted lines. Finally, we subtracted this amplitude from the waveform peak and divided this difference (A in Fig. 2c) by the pulse pressure (PP, i.e., B in Fig. 2c). Like the carotid augmentation index^29^, this carotid upstroke index (CUI) may not only increase with aortic diameter but also age and BP. Hence, we normalized the CUI as CUI/(age·DBP). This feature may increase with the aortic diameter.

We extracted the popular features from the carotid BP waveform (see Fig. 3). These features have been extensively studied for other applications^30,31^ and comprise time intervals, amplitudes, and areas of the waveform and its first and second derivatives.

#### Model development

We employed powerful linear regression to predict the maximal aortic diameter from the waveform features. We sought to develop models that could predict the maximal aortic diameter over its physiologic range from normal to severely diseased. Although we only had the image-based reference maximal aortic diameter measurements for the AAA patients, we did have strong pre-knowledge of the quantitative characteristics of the maximal aortic diameters for the control patients. In particular, the mean and standard deviation of the normal abdominal aortic diameter at the level of the renal arteries are 2.2 and 0.4 cm^11^. We thus modeled the reference maximal aortic diameter measurements for the control patients using Gaussian white noise with these statistics. This modeling of the control reference values may also be rationale in that it may not be possible to discriminate the relatively narrow range of maximal aortic diameters for control patients via arterial waveform analysis anyhow. Instead, our specific hypothesis is that arterial waveform analysis can discriminate larger maximal aortic diameter changes that occur with AAA growth and treatment.

We developed models to predict the measured or modeled reference maximal aortic diameters from all waveform features (carotid+femoral feature model) or from carotid waveform features alone (carotid feature model). For each model, we also allowed demographics, risk factors, and basic hemodynamic values (i.e., all variables in Table 1 except for maximal aortic diameter) as possible non-waveform features. For comparison, we developed a third linear regression model to predict the maximal aortic diameter from the non-waveform features alone. This baseline model was important due to the imperfect matching of the AAA and control patients (see Table 1). We adjusted each continuous feature to zero-mean and unit-variance and included each binary categorical variable as two model intercepts (as opposed to model slopes or coefficients) corresponding to each category. Each model also included an overall intercept equal to the average maximal aortic diameter in the training data.

We selected the features and determined the regression parameters (coefficients and intercepts) similar to our prior work^21^. More specifically, because our patient sample size was not large, we employed leave-one-patient-out cross validation to use as much data as possible for training while also allowing testing on all patients without using the same data for training and testing. For each model, we trained 100 regression equations using the measured or modeled reference maximal aortic diameters (dependent variable) and waveform and non-waveform features (independent variables) from all combinations of 99 of the AAA (pre-EVAR) and control patients and left the remaining patient of each combination for testing. For each of the 100 training sets, we applied forward stepwise regression in conjunction with an “elbow” method to determine the number of features by penalizing for model complexity. We added one feature at a time to the regression equation, starting with zero features and ending with five features, and selected the additional feature at each iteration as the one that yielded the minimum mean-squared maximal aortic diameter prediction error. We set the maximal number of features to five, because about 20 datapoints per parameter are usually needed. We thus created six optimal equations corresponding to 0 to 5 features. We then fitted two lines to the monotonically decreasing curve relating the minimum mean-squared prediction error to the number of features and determined the number of features, and thus the final regression equation, via the intersection of the two lines (i.e., the curve’s elbow). Our experience has been that this simple method produces more parsimonious and stable models than more popular methods such as Akaike’s or Bayesian Information Criteria minimization.

#### Model evaluation

We evaluated the 100 leave-one-patient-out maximal aortic diameter predictions of each of the three models in two ways to ascertain “AAA screening” capabilities. First, we assessed the accuracy of the predicted maximal aortic diameters of each model against the measured or modeled reference maximal aortic diameters using correlation and Bland-Altman analyses (see second to last paragraph of Discussion section for rationale). We statistically compared the (i) R value for each waveform feature model with that for the baseline model using a common test for dependent data^12^ and (ii) NRMSE (=√(μ^2^ + σ^2^), where μ and σ are the conventional bias and precision errors, divided by the average of the 100 reference image-based maximal aortic diameters and given in percent) for each waveform feature model with that for the baseline model using nonparametric bootstrapping for paired data^21,32^. Second, we evaluated the predicted maximal aortic diameters of each model in terms of their ability to classify AAA versus control patients using ROC curve analysis. We statistically compared the ROC AUC for each waveform feature model with that for the baseline model using the Hanley–McNeil test for paired data^33^. ROC curve analysis quantifies the ability to classify individuals, and the AUC indicates the probability of correctly classifying two individuals wherein the individuals are from different classes. This analysis is more relevant than common *t*- and U-tests, which quantify the ability to classify groups of individuals. We smoothed the ROC curves without impacting the AUCs to be able to reliably estimate sensitivity at a given specificity^34^.

We also evaluated each of the three models in terms of their ability to track the maximal aortic diameter following the EVAR to assess “AAA surveillance” capabilities. Since reference image-based maximal aortic diameter measurements were not available post-EVAR, we leveraged knowledge that successful surgery will substantially reduce the effective maximal aortic diameter. For each model, we first applied a regression equation trained using all data from the 100 AAA (pre-EVAR) and control patients to predict the maximal aortic diameter from the waveform or non-waveform features post-EVAR. We then employed ROC curve analysis to assess the out-of-training predicted maximal aortic diameters of each model in terms of their ability to classify AAA patients before versus 4 weeks after EVAR. We likewise assessed the predicted maximal aortic diameter changes of each model in terms of their ability to classify the change from before to 4 weeks after EVAR versus the change from 4 weeks to 3 years post-EVAR. This second classification task assumed that the maximal aortic diameter changed appreciably more following EVAR than 3 years of aging. We again used the Hanley–McNeil test to compare the ROC AUCs of the models for each of the classification tasks.

We thus employed three evaluation metrics (R, NRMSE, and AUC) and performed statistical comparisons against a baseline model. This approach, along with the patient matching, mitigated the possibility of obtaining trivial results.

#### Preliminary analysis

Prior to performing the aforementioned model development and evaluation, we developed the linear regression models using the data from all 50 AAA and 50 control patients for 100 different realizations of the Gaussian white noise model of the reference maximal aortic diameters for the control patients. We found that the selected features for the carotid+femoral and carotid feature models were the same for 94 and 100 of the realizations and that the coefficients of the models with the common input features had a coefficient of variation of only <5%. Due to this consistency, we then performed the aforementioned model development and evaluation for one realization of the Gaussian white noise to conveniently present representative results.

### Reporting summary

Further information on research design is available in the Nature Research Reporting Summary linked to this article.

## Supplementary information

Reporting Summary

## Data Availability

The data described in this manuscript may be made available upon reasonable request to H.-M.C. (hmcheng@vghtpe.gov.tw).

## Code availability

The code may be made available upon reasonable request to R.M. (rmukkamala@pitt.edu).

## References

[1] Aggarwal S, Qamar A, Sharma V, Sharma A (2011). Abdominal aortic aneurysm: a comprehensive review. Exp. Clin. Cardiol..

[2] LeFevre ML (2014). Screening for abdominal aortic aneurysm: U.S. preventive services task force recommendation statement. Ann. Intern. Med..

[3] Chaikof E (2018). The society for vascular surgery practice guidelines on the care of patients with an abdominal aortic aneurysm. J. Vasc. Surg..

[4] Smith-Burgess L. *Early identification and detection of abdominal aortic aneurysms | Clinical | Nursing Times. Nursing Times*. https://www.nursingtimes.net/clinical-archive/cardiovascular-clinical-archive/early-identification-and-detection-of-abdominal-aortic-aneurysms-27-02-2017/ (2017).

[5] Zucker EJ, Prabhakar AM (2018). Abdominal aortic aneurysm screening: concepts and controversies. Cardiovasc. Diagn. Ther..

[6] Olchanski N, Winn A, Cohen JT, Neumann PJ (2014). Abdominal aortic aneurysm screening: how many life years lost from underuse of the medicare screening benefit?. J. Gen. Intern. Med..

[7] Brady AR, Thompson SG, Fowkes FGR, Greenhalgh RM, Powell JT (2004). Abdominal aortic aneurysm expansion: Risk factors and time intervals for surveillance. Circulation.

[8] Zucker EJ, Misono AS, Prabhakar AM (2017). Abdominal aortic aneurysm screening practices: impact of the 2014 U.S. preventive services task force recommendations. J. Am. Coll. Radiol..

[9] Yu WC, Chuang SY, Lin YP, Chen CH (2008). Brachial-ankle vs carotid-femoral pulse wave velocity as a determinant of cardiovascular structure and function. J. Hum. Hypertens..

[10] Lee CW (2013). Measures of carotid-femoral pulse wave velocity and augmentation index are not reliable in patients with abdominal aortic aneurysm. J. Hypertens..

[11] Creager, M. *Screening for abdominal aortic aneurysm*. https://www.uptodate.com/contents/screening-for-abdominal-aortic-aneurysm (2022).

[12] Lenhard, W. & Lenhard, A. *Hypothesis Tests for Comparing Correlations.* Bibergau (Germany): Psychometrica. 10.13140/RG.2.1.2954.1367 (2014).

[13] Swillens A (2008). Effect of an abdominal aortic aneurysm on wave reflection in the aorta. IEEE Trans. Biomed. Eng..

[14] Bailey M (2014). Carotid-femoral pulse wave velocity is negatively correlated with aortic diameter. Hypertens. Res..

[15] Tavallali P, Razavi M, Pahlevan NM (2018). Artificial intelligence estimation of carotid-femoral pulse wave velocity using carotid waveform. Sci. Rep..

[16] Martin SLO (2016). Weighing scale-based pulse transit time is a superior marker of blood pressure than conventional pulse arrival time. Sci. Rep..

[17] Inan O, Etemadi M, Wiard R, Giovangrandi L, Kovacs G (2009). Robust ballistocardiogram acquisition for home monitoring. Physiol. Meas..

[18] Kim C-S, Carek AM, Inan O, Mukkamala R, Hahn J-O (2018). Ballistocardiogram-based approach to cuff-less blood pressure monitoring: proof-of-concept and potential challenges. IEEE Trans. Biomed. Eng..

[19] Kim CS (2016). Ballistocardiogram: mechanism and potential for unobtrusive cardiovascular health monitoring. Sci. Rep..

[20] Nichols, W. W., O’Rourke, M. & Vlachopoulos, C. Pressure Pulse Waveform Analysis. in *McDonald’s Blood Flow in Arteries: Theoretical, Experimental and Clinical Principles* 630 (CRC press, 2011).

[21] Natarajan K (2022). Photoplethysmography fast upstroke time intervals can be useful features for cuff-less measurement of blood pressure changes in humans. IEEE Trans. Biomed. Eng..

[22] Chiu Y, Arand P, Shroff S, Feldman T, Carroll J (1991). Determination of pulse wave velocities with computerized algorithms. Am. Heart J..

[23] Nichols, W. W., O’Rourke, M. & Vlachopoulos, C. Central Arterial Pressure. in *McDonald’s Blood Flow in Arteries: Theoretical, Experimental and Clinical Principles* 575 (CRC press, 2011).

[24] Xiong J, Wang SM, Zhou W, Wu JG (2008). Measurement and analysis of ultimate mechanical properties, stress-strain curve fit, and elastic modulus formula of human abdominal aortic aneurysm and nonaneurysmal abdominal aorta. J. Vasc. Surg..

[25] Koullias G (2005). Mechanical deterioration underlies malignant behavior of aneurysmal human ascending aorta. J. Thorac. Cardiovasc. Surg..

[26] Mattace-Raso FUS (2010). Determinants of pulse wave velocity in healthy people and in the presence of cardiovascular risk factors: ‘Establishing normal and reference values’. Eur. Heart J..

[27] Mukkamala R (2015). Toward ubiquitous blood pressure monitoring via pulse transit time: theory and practice. IEEE Trans. Biomed. Eng..

[28] Zhang G, Hahn JO, Mukkamala R (2011). Tube-load model parameter estimation for monitoring arterial hemodynamics. Front. Physiol..

[29] Fantin F, Mattocks A, Bulpitt CJ, Banya W, Rajkumar C (2007). Is augmentation index a good measure of vascular stiffness in the elderly?. Age Ageing.

[30] Elgendi M (2012). On the analysis of fingertip photoplethysmogram signals. Curr. Cardiol. Rev..

[31] Addison P (2016). Slope transit time (STT): a pulse transit time proxy requiring only a single signal fiducial point. IEEE Trans. Biomed. Eng..

[32] Efron, B. & Tabshirani, R. The Bootstrap Estimate of Standard Error. in *An Introduction to the Bootstrap* (CRC Press, 1993).

[33] Hanley JA, McNeil BJ (1983). A method of comparing the areas under receiver operating characteristic curves derived from the same cases. Radiology.

[34] Zou K, Hall W, Shapiro D (1997). Smooth non‐parametric receiver operating characteristic (ROC) curves for continuous diagnostic tests. Stat. Med..

