# Supplementary material for: Abdominal aortic aneurysm monitoring via arterial waveform analysis: towards a convenient point-of-care device
Source: NPJ Digit Med. 2022 Nov 4;5:168. doi: 10.1038/s41746-022-00717-3 (PMC9633589; doi:10.1038/s41746-022-00717-3)
Supplement: Supplementary file 1 — Reporting Summary [file 41746_2022_717_MOESM1_ESM.pdf]

nature portfolio

Corresponding author(s): Mukkamala, Ramakrishna

Last updated by author(s): Jul 25, 2022

Reporting Summary

Nature Portfolio wishes to improve the reproducibility of the work that we publish. This form provides structure for consistency and transparency in reporting. For further information on Nature Portfolio policies, see our Editorial Policies and the Editorial Policy Checklist.

Statistics

For all statistical analyses, confirm that the following items are present in the figure legend, table legend, main text, or Methods section.

n/a	Confirmed	
☐	☒	The exact sample size (*n*) for each experimental group/condition, given as a discrete number and unit of measurement
☐	☒	A statement on whether measurements were taken from distinct samples or whether the same sample was measured repeatedly
☐	☒	The statistical test(s) used AND whether they are one- or two-sided<br>*Only common tests should be described solely by name; describe more complex techniques in the Methods section.*
☐	☒	A description of all covariates tested
☐	☒	A description of any assumptions or corrections, such as tests of normality and adjustment for multiple comparisons
☐	☒	A full description of the statistical parameters including central tendency (e.g. means) or other basic estimates (e.g. regression coefficient) AND variation (e.g. standard deviation) or associated estimates of uncertainty (e.g. confidence intervals)
☐	☒	For null hypothesis testing, the test statistic (e.g. *F*, *t*, *r*) with confidence intervals, effect sizes, degrees of freedom and *P* value noted<br>*Give P values as exact values whenever suitable.*
☒	☐	For Bayesian analysis, information on the choice of priors and Markov chain Monte Carlo settings
☒	☐	For hierarchical and complex designs, identification of the appropriate level for tests and full reporting of outcomes
☐	☒	Estimates of effect sizes (e.g. Cohen's *d*, Pearson's *r*), indicating how they were calculated

Our web collection on statistics for biologists contains articles on many of the points above.

Software and code

Policy information about availability of computer code

Data collection	No data were collected in this secondary analysis of existing, de-identified patient data.
Data analysis	Custom code was created to analyze the data using the MATLAB software package (R 2019a).

For manuscripts utilizing custom algorithms or software that are central to the research but not yet described in published literature, software must be made available to editors and reviewers. We strongly encourage code deposition in a community repository (e.g. GitHub). See the Nature Portfolio guidelines for submitting code & software for further information.

Data

Policy information about availability of data

All manuscripts must include a data availability statement. This statement should provide the following information, where applicable:

- Accession codes, unique identifiers, or web links for publicly available datasets
- A description of any restrictions on data availability
- For clinical datasets or third party data, please ensure that the statement adheres to our policy

The data presented in this manuscript may be made available upon reasonable request to Hao-min Cheng (hmcheng@vghtpe.gov.tw).

Human research participants

Policy information about studies involving human research participants and Sex and Gender in Research.

Reporting on sex and gender	This study concerns abdominal aortic aneurysms, which is primarily a male disease (see second paragraph in Introduction). The study was also a secondary analysis of existing, de-identified patient data, and males were most prevalent in the database.
Population characteristics	The study population included patients with abdominal aortic aneurysms. These patients were predominantly male and of advanced age, as these are risk factors for the disease. The study population also included matched control patients without any aortic aneurysm.
Recruitment	This study was a secondary analysis of existing, de-identified patient data.
Ethics oversight	The University of Pittsburgh's IRB declared that this secondary analysis of the existing, de-identified patient database met the regulatory requirements for exempt research (STUDY22020004).

Note that full information on the approval of the study protocol must also be provided in the manuscript.

Field-specific reporting

Please select the one below that is the best fit for your research. If you are not sure, read the appropriate sections before making your selection.

☒ Life sciences ☐ Behavioural & social sciences ☐ Ecological, evolutionary & environmental sciences

For a reference copy of the document with all sections, see nature.com/documents/nr-reporting-summary-flat.pdf

Life sciences study design

All studies must disclose on these points even when the disclosure is negative.

Sample size	We did not have preliminary data for a credible a priori sample size calculation. This study was also a secondary analysis of existing, de-identified patient data. We extracted and much relevant patient data as possible. We obtained statistically significant results, and the p-values were not tiny. So, the study appeared appropriately powered in post-hoc analysis.
Data exclusions	Our pre-defined data exclusion criteria were (i) multiple aortic aneurysms; (ii) peripheral arterial disease (PAD) as ascertained via an ankle-brachial index (ABI) < 0.9, (iii) non-sinus rhythm; and (iv) artifact-contaminated tonometric waveforms based on visual inspection. Another criteria was to match the abdominal aortic aneurysm patients with control patients in terms of non-waveform characteristics. This matching was crucial for mitigating the possibility of obtaining trivial results.
Replication	We used cross validation to maximize the likelihood of reproducibility. This is the standard approach in machine learning.
Randomization	We used leave-one-patient-out cross validation, so randomization was not a factor in assigning patients to the training and testing groups.
Blinding	Cross validation is an approach for the investigators to be appropriately blinded to the testing data.

Reporting for specific materials, systems and methods

We require information from authors about some types of materials, experimental systems and methods used in many studies. Here, indicate whether each material, system or method listed is relevant to your study. If you are not sure if a list item applies to your research, read the appropriate section before selecting a response.

Materials & experimental systems

n/a	Involved in the study	
☒	☐	Antibodies
☒	☐	Eukaryotic cell lines
☒	☐	Palaeontology and archaeology
☒	☐	Animals and other organisms
☒	☐	Clinical data
☒	☐	Dual use research of concern

Methods

n/a	Involved in the study	
☒	☐	ChIP-seq
☒	☐	Flow cytometry
☒	☐	MRI-based neuroimaging
